# The effect of medical comorbidity on HOOS/KOOS/FAOS: a national register-based cohort study of 7850 representative citizens

**DOI:** 10.1007/s10067-025-07372-5

**Published:** 2025-02-22

**Authors:** Peter Larsen, Rasmus Elsoe

**Affiliations:** 1 Department of Orthopaedic Trauma Surgery, Aalborg University Hospital, 18-22 Hobrovej, DK-9000 Aalborg, Denmark; 2 Department of Occupational Therapy and Physiotherapy, Aalborg University Hospital, Aalborg, Denmark

**Keywords:** Comorbidity, HOOS/KOOS/FAOS, Outcome, PROM

## Abstract

**Objectives:**

Despite extensive validation, the impact of medical comorbidities on the outcomes of the Hip Disability and Osteoarthritis Outcome Score (HOOS), Knee Injury Osteoarthritis Outcome Score (KOOS), and Foot and Ankle Outcome Score (FAOS) remains underexplored. This study aimed to evaluate the effect of medical comorbidities on HOOS, KOOS, and FAOS subscales using a large, nationally representative sample.

**Methods:**

This national register-based cohort study invited 26,877 participants to complete HOOS, KOOS, or FAOS questionnaires. Medical comorbidities—including diabetes, chronic obstructive pulmonary disease/asthma, rheumatological diseases, osteoporosis, stroke, obesity, and heart disease—were identified through the Danish National Patient Register.

**Results:**

A total of 7850 participants (29%) responded, with 1863 (24%) having medical comorbidities. HOOS/KOOS/FAOS subscale scores were significantly worse in patients with comorbidities, particularly in the Sport/Rec, ADL, and QOL subscales. Mean score differences between participants with and without comorbidities were pain (− 5.7, 95% CI − 6.6 to − 4.7), symptoms (− 4.6, 95% CI − 5.5 to − 3.6), ADL (− 7.1, 95% CI − 8.0 to − 6.1), Sport/Rec (− 10.4, 95% CI − 11.9 to − 8.9), and QOL (− 6.9, 95% CI − 8.2 to − 5.7). Diabetes, rheumatological diseases, and obesity were associated with the greatest complaints.

**Conclusion:**

Patients with medical comorbidity reported significantly lower HOOS/KOOS/FAOS subscale scores compared to participants without medical comorbidity. Diabetes, chronic rheumatological diseases, and adiposities were observed with the most complaints.

**Table:** 

**Key Points** • *Medical comorbidity predicts considerably lower HOOS/KOOS/FAOS subscale scores.* • *Diabetes, rheumatological diseases, and obesity exerted the most pronounced negative effects on the HOOS/KOOS/FAOS.* • *Findings underscore the importance of considering comorbidities when interpreting HOOS/KOOS/FOAS subscale scores.*

**Supplementary Information:**

The online version contains supplementary material available at 10.1007/s10067-025-07372-5.

## Introduction

Patient-reported outcome measures (PROMs) are widely used in musculoskeletal clinical practice to assess patient-perceived health status and monitor changes following conservative, medical, or surgical treatment [1, 2]. In contrast to generic PROMs, body-region-specific measurements provide targeted insights into pain, function, and quality of life for specific anatomic regions such as the hip, knee, or foot/ankle [2].

A widely used set of body-region-specific PROMs for the lower limbs are the Hip Disability and Osteoarthritis Outcome Score (HOOS) [3], the Knee Injury Osteoarthritis Outcome Score (KOOS) [4], and the Foot and Ankle Outcome Score (FAOS) [5]. The KOOS questionnaire was originally developed in 1995 by Professor Ewa M. Roos and colleagues at the Departments of Orthopedics at Lund University, Sweden [4]. The KOOS was later modified to other body regions such as the hip (HOOS) and foot/ankle conditions (FAOS).

Interpretation of the HOOS/KOOS/FAOS has been improved during extensive validation and by providing large-scale national representative reference values [5–11]. Current literature has mostly focused on psychometric properties across musculoskeletal conditions and treatment outcomes [11]. Age and sex have been reported with low effect of outcome, in contrast to body mass index and osteoarthritis reported with a considerable effect [8–10]. Despite extensive validation, the influence of medical comorbidities on the HOOS/KOOS/FAOS is poorly understood [12]. It is well-known that many chronic and long-term medical comorbidities such as diabetes, chronic obstructive pulmonary disease, and cardiovascular diseases are reported with significant association to worse musculoskeletal health [13]. Understanding the specific effects of medical comorbidities will likely improve the interpretation of the HOOS/KOOS/FAOS in both clinical and research settings [14].

This study aimed to investigate the effect of medical comorbidity on the HOOS, KOOS, and FAOS five subscales based on a large-scale national representative sample.

## Methods

### Study design

This study utilized a national register-based cohort design investigating medical comorbidity on the HOOS/KOOS/FAOS. Medical comorbidities were predefined using seven diagnostic categories: diabetes, chronic obstructive pulmonary disease (COPD) or asthma, chronic rheumatological diseases, osteoporosis, stroke, adiposities, and heart disease.

The Danish Data Protection Agency approved the study (J. nr. 2021 Id: 114). The reporting of the study complies with the Strengthening the Reporting of Observational Studies in Epidemiology (STROBE) Statement [15].

### Data retrieval

At birth or immigration to Denmark, a civil registration number (CPR) is given to all residents and registered in the Civil Registration System [16]. Prospective information regarding emigration and death is recorded in this registry [16]. The Civil Registration System includes individual information on the complete population of Denmark [16].

**Fig. 1:**
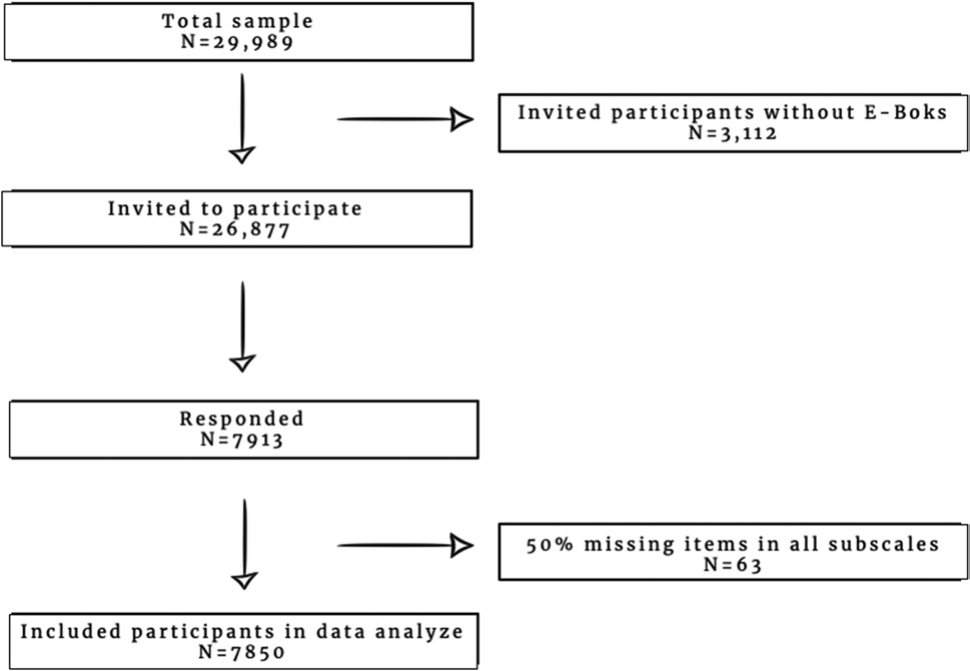
Flow of the study

Included was a representative sample of 29,989 unique adult citizens of Denmark. The population was derived from the Danish Civil Registration System, selected to be representative for the Danish adult population on age and gender. Excluded were citizens without online contact information (E-boks). E-boks is mandatory for almost all adult Danish citizens and is connected to the Civil Registration System (Fig. 1).

### Study population/survey invitation

Following the exclusion of citizens without E-boks, the representative sample consisted of 26,877 citizens who were invited to participate in the study by June 2022. Non-responders received a second and final invitation after 14 days.

### The HOOS, KOOS, and FAOS

The HOOS, KOOS, and FAOS are patient-reported body-region-specific questionnaires including 40–42 items in five subscales evaluating pain, symptoms, function of daily living (ADL), function in sport and recreation (Sport/Rec), and quality of life (QOL) [4]. The HOOS, KOOS, and FAOS are developed to assess the patients’ opinion regarding their hip, knee, or ankle/foot and associated problems [4]. The HOOS, KOOS, and FAOS have been validated for several musculoskeletal conditions related to the hip, knee, and ankle/foot and have been cross-culturally adapted into several languages.

The outcome is calculated based on a standardized scoring algorithm with a score between 0 and 100 for each of the five subscales [4]. A score of 100 indicates the best possible results and 0 the worst outcome [4]. The questionnaires are freely available for academic users at https://eprovide.mapi-trust.org.

### Medical comorbidity

Danish law requires that all patient contacts with hospital and outpatient clinics be registered in the Danish National Patient Register [16]. Date, time of activity, diagnoses, and procedures are registered on an individual level by using the CPR. This enables a complete health registration of the total population of Denmark.

Medical comorbidities on an individual level were identified through the Danish National Patient Register, which records all hospital and outpatient contacts through the CPR. Diagnoses were classified into predefined categories (diabetic, COPD or asthma, chronic rheumatological diseases, osteoporosis, stroke, adiposity, and heart disease) using ICD-9 and ICD-10 codes (see Supplementary Table 1).

### Statistical analysis

Continuous variables are reported by mean and standard deviation ( ±) or 95% confidence intervals (CI) and categorical variables by frequencies. Pooled HOOS, KOOS, and FAOS subscale scores were analyzed and further supplemented by HOOS, KOOS, and FAOS subscale scores.

The ANOVA test was used to analyze the effect of multi-disease on the pooled HOOS, KOOS, and FAOS subscale scores. For analysis, three groups were established: a group with one comorbidity, a group with two comorbidities, and a group with three or more comorbidities. The Bonferroni post hoc test was used for multiple comparisons.

Multivariable linear regression analyses were performed to analyze the effect of the selected medical comorbidity diagnoses on the pooled HOOS, KOOS, and FAOS subscale scores.

## Results

Of the 26,877 citizens invited, 7850 (29%) responded to the survey. Among respondents, 1863 individuals (24%) had at least one medical comorbidity, distributed as follows: one comorbidity (*n* = 1152), two comorbidities (*n* = 562), and three or more comorbidities (*n* = 139). The remaining 5987 participants (76%) had no recorded comorbidities.

The most prevalent conditions were heart disease (14%), COPD/asthma (8%), and diabetes (4%), followed by stroke (6%), rheumatological diseases (2%), osteoporosis (1%), and obesity (0.5%).

The mean age of citizens presenting with medical comorbidity was 68.2 years, and 45% were of female gender, compared to citizens without medical comorbidity presenting with a mean age of 58.0 years and 57% female gender. The mean time from comorbidity diagnoses to follow-up (time from diagnoses to completing HOOS, KOOS, or FAOS) was 14.4 (SD 8.0) years, ranging from 3.3 to 44.9 years.

Detailed information of the study population is presented in Table 1 and Fig. 1.

**Table 1:** Baseline characteristics of 7850 citizens

	All	+ comorbidity	− comorbidity
Age at survey, years (SD)	60.4 (16.6)	68.2 (14.4)	58.0 (16.5)
Gender, women/men	4281/3569	843/1020	3429/2558
Height, m (SD)	1.72 (9.1)	1.73 (8.9)	1.73 (9.2)
Weight, kg (SD)	79.9 (18.1)	82.2 (18.7)	79.2 (17.8)
BMI (SD)	26.6 (4.9)	27.6 (5.6)	26.5 (5.3)

*BMI* body mass index

### The effect of medical comorbidity on HOOS/KOOS/FAOS

Participants with medical comorbidities reported significantly lower scores across all subscales of HOOS, KOOS, and FAOS compared to participants without comorbidities. The largest differences were observed in the subscales Sport/Rec, ADL, and QOL. The mean score differences (pooled for HOOS/KOOS/FAOS) between participants with and without comorbidities were pain − 5.7 (95% CI − 6.6 to − 4.7), symptoms − 4.6 (95% CI − 5.5 to − 3.6), ADL − 7.1 (95% CI − 8.0 to − 6.1), Sport/Rec − 10.4 (95% CI − 11.9 to − 8.9), and QOL − 6.9 (95% CI − 8.2 to − 5.7) (Table 2).

**Table 2:** HOOS, KOOS, and FAOS mean subscale scores and 95% CI, divided by patients with and without comorbidity

	All (HOOS + KOOS + FAOS)			HOOS			KOOS			FAOS		
	+ comorbidity	− comorbidity	Mean diff 95% CI	+ comorbidity	− comorbidity	Mean diff 95% CI	+ comorbidity	− comorbidity	Mean diff 95% CI	+ comorbidity	− comorbidity	Mean diff 95% CI
Pain	82.0 (81.0–83.0)	87.7 (87.2–88.1)	− 5.7 (− 6.6 to − 4.7)	81.1 (79.2–82.9)	88.7 (87.8–89.5)	− 7.6 (− 9.5 to − 5.7)	82.7 (81.2–84.2)	86.0 (85.3–86.7)	− 3.3 (− 4.9 to − 1.7)	82.1 (80.4–83.7)	88.6 (87.8–89.3)	− 6.5 (− 8.1 to − 4.9)
Symptoms	81.1 (80.2–82.0)	85.7 (85.2–86.1)	− 4.6 (− 5.5 to − 3.6)	78.5 (76.7–80.3)	85.2 (84.3–86.1)	− 6.7 (− 8.6 to − 4.8)	84.5 (83.2–85.8)	85.4 (84.7–86.1)	− 0.9 (− 0.2 to 0.6)	80.0 (78.5–81.6)	86.5 (85.8–87.2)	− 6.5 (− 8.0 to − 5.0)
ADL	82.5 (81.5–83.4)	89.5 (89.1–90.0)	− 7.1 (− 8.0 to − 6.1)	82.3 (80.5–84.2)	90.1 (89.2–90.9)	− 7.7 (− 9.6 to − 5.9)	82.8 (81.3–84.3)	87.8 (87.1–88.5)	− 5.1 (− 6.6 to − 3.5)	82.2 (80.5–83.9)	90.8 (90.1–91.5)	− 8.6 (− 10.2 to − 7.1)
Sport/Rec	69.0 (67.5–70.5)	79.4 (78.7–80.1)	− 10.4 (− 11.9 to − 8.9)	73.7 (71.2–76.3)	85.9 (84.8–87.0)	− 12.1 (− 14.5 to − 9.7)	65.2 (62.7–67.7)	72.6 (71.3–73.8)	− 7.4 (− 10.0 to − 4.7)	69.0 (66.3–71.6)	81.3 (80.2–82.5)	− 12.4 (− 14.9 to − 9.8)
QOL	75.5 (72.3–74.8)	80.5 (79.9–81.1)	− 6.9 (− 8.2 to − 5.7)	76.7 (74.6–78.9)	84.5 (83.4–85.5)	− 7.7 (− 10.0 to − 5.5)	71.7 (69.7–73.8)	75.8 (74.8–76.9)	− 4.1 (− 6.3 to − 1.9)	72.7 (70.5–74.9)	82.0 (81.0–83.0)	− 9.3 (− 11.5 to − 7.1)

*N* number, *CI* confidence interval

Further subgroup analysis comparing medical comorbidity separately to HOOS, KOOS, and FAOS showed comparable results with considerable complaints for all three questionnaires (Table 3).

**Table 3:** Pooled HOOS/KOOS/FAOS mean subscale scores and 95% CI, divided by the seven comorbidity groups

	Heart disease *N* = 1113			Pulmonary disease *N* = 607			Rheumatological disease *N* = 151			Osteoporosis *N* = 54		
	+ heart disease	− heart disease	Mean diff 95% CI	+ pulmonary disease	− pulmonary disease	Mean diff 95% CI	+ rheumatological diseases	− rheumatological diseases	Mean diff 95% CI	+ osteoporosis	− osteoporosis	Mean diff 95% CI
Pain	83.4 (82.2–84.6)	86.8 (86.4–87.3)	− 3.5 (− 4.7 to − 2.3)	80.7 (79.0–82.4)	86.8 (86.4–87.2)	− 6.1 (− 7.6 to − 4.5)	76.1 (72.4–79.9)	86.5 (86.1–86.9)	− 10.4 (− 13.4 to − 7.3)	78.6 (72.1–85.0)	86.4 (86.0–86.8)	− 7.8 (− 12.9 to − 2.8)
Symptoms	82.9 (81.8–84.0)	84.9 (84.5–85.3)	− 2.0 (− 3.2 to − 0.9)	79.2 (77.6–80.8)	85.1 (84.7–85.5)	− 5.9 (− 7.4 to − 4.4)	75.5 (72.0–79.0)	84.8 (84.4–85.2)	− 9.3 (− 12.1 to − 6.4)	81.6 (76.5–87.2)	84.6 (84.2–85.0)	− 2.8 (− 7.6 to 2.0)
ADL	83.0 (81.8–84.3)	88.7 (88.2–89.1)	− 5.6 (− 6.8 to − 4.5)	82.2 (80.5–83.9)	88.3 (87.9–88.8)	− 6.1 (− 7.7 to − 4.7)	76.8 (73.2–80.5)	88.1 (87.7–88.5)	− 11.2 (− 14.2 to − 8.3)	77.1 (70.8–83.5)	87.9 (87.5–88.3)	− 10.8 (− 15.6 to − 6.0)
Sport/Rec	70.4 (68.5–72.2)	78.1 (77.4–78.8)	− 7.7 (− 9.6 to − 5.9)	68.8 (66.2–71.4)	77.7 (77.0–78.3)	− 8.9 (− 11.3 to − 6.4)	59.2 (53.5–64.9)	77.3 (76.7–78.0)	− 18.1 (− 22.9 to − 13.4)	64.9 (54.8–74.9)	77.1 (76.4–77.7)	− 12.2 (− 20.3 to − 4.1)
QOL	75.3 (73.7–76.8)	79.4 (78.8–80.0)	− 4.1 (− 5.7 to − 2.6)	72.5 (70.4–74.7)	79.4 (78.8–79.9)	− 6.9 (− 8.9 to − 4.8)	65.1 (60.5–69.7)	79.1 (78.6–79.7)	− 14.0 (− 18.0 to − 10.0)	69.3 (61.3–77.0)	78.9 (78.4–79.5)	− 9.6 (− 16.3 to − 2.9)
	Stroke *N* = 443			Adipositas *N* = 34			Diabetes *N* = 314					
	+ stroke	− stroke	Mean diff 95% CI	+ adipositas	− adipositas	Mean diff 95% CI	+ diabetes	− diabetes	Mean diff 95% CI			
Pain	81.7 (79.7–83.7)	86.6 (86.2–87.0)	− 4.9 (− 6.8 to − 3.1)	70.4 (61.8–78.9)	86.4 (86.0–86.8)	− 16.0 (− 22.3 to − 9.7)	77.2 (74.7–79.7)	86.7 (86.3–87.1)	− 9.5 (− 11.6 to − 7.4)			
Symptoms	81.0 (79.1–82.9)	84.8 (84.4–85.2)	− 3.9 (− 5.6 to − 2.1)	74.4 (67.2–81.7)	84.7 (84.3–85.1)	− 10.3 (− 16.3 to − 4.2)	76.5 (74.0–78.9)	85.0 (84.6–85.4)	− 8.5 (− 10.5 to − 6.5)			
ADL	81.2 (79.2–83.2)	88.3 (87.9–88.7)	− 7.1 (− 8.8 to − 5.3)	71.5 (63.4–79.5)	87.9 (87.5–88.3)	− 16.5 (− 22.7 to − 10.3)	76.8 (74.2–79.4)	88.3 (87.9–88.7)	− 11.6 (− 13.6 to − 9.5)			
Sport/Rec	68.3 (65.2–71.3)	77.5 (76.8–78.2)	− 9.2 (− 12.1 to − 6.4)	55.7 (43.6–67.9)	77.1 (76.4–77.1)	− 21.3 (− 31.5 to − 11.1)	61.0 (57.1–64.8)	77.7 (77.0–78.3)	− 16.7 (− 20.0 to − 13.4)			
QOL	73.5 (71.0–76.1)	79.2 (78.6–79.7)	− 5.7 (− 8.0 to − 3.3)	66.8 (57.3–76.2)	78.9 (78.4–79.5)	− 12.1 (− 20.6 to − 3.7)	67.7 (64.5–70.8)	79.3 (78.8–79.9)	− 11.6 (− 14.4 to − 8.9)			

*CI* confidence interval, *N* number

Further analysis adjusted for age and sex on the pooled HOOS/KOOS/FAOS subscale scores showed pain − 5.8 (95% CI − 6.8 to − 4.8), symptoms − 5.3 (95% CI − 6.3 to − 4.4), ADL − 6.2 (95% CI − 7.2 to − 5.4), Sport/Rec − 8.7 (95% CI − 10.3 to − 7.2), and QOL − 6.8 (95% CI − 8.2 to − 5.6).

### The effect of selected comorbidity groups on the pooled HOOS/KOOS/FAOS scores

The effects of diabetes, COPD or asthma, chronic rheumatological diseases, osteoporosis, stroke, adiposities, and heart disease are presented in Table 3. Diabetes, rheumatological diseases, and adiposities were observed with most complaints on the HOOS, KOOS, and FAOS. These results were also evident when applying subscale scores to a stepwise multiple regression model (Table 4).

**Table 4:** Effect of selected comorbidity groups on the pooled HOOS/KOOS/FAOS scores

Crude regression					
Crude	Pain	Symptoms	ADL	Sport/Rec	QOL
Heart disease	− 3.5, *p* < 0.00	− 2.0, *p* < 0.00	− 5.6, p < 0.00	− 7.7, *p* < 0. − 0	− 4.1, *p* < 0.00
Pulmonary disease	− 6.1, *p* < 0.00	− 5.9, *p* < 0.00	− 6.2, *p* < 0.00	− 8.9, *p* < 0.00	− 6.9, *p* < 0.00
Reuma	− 10.4 *p* < 0.00	− 9.3, *p* < 0.00	− 11.2, *p* < 0.00	− 18.1, *p* < 0.00	− 14.0, *p* < .00
Osteoporosis	− 2.8, p 0.25	− 7.8, *p* 0.002	− 10.8, *p* < 0.00	− 12.2, *p* 0.003	− 9.6, *p* 0.005
Stroke	− 4.9 *p* < 0.00	− 3.9, *p* < .0.00	− 7.1, *p* < 0.00	− 9.2, *p* < 0.00	− 5.7, *p* < 0.00
Diabetic disease	− 9.5, *p* < 0.00	− 8.5, *p* 0.00	− 11.6, *p* < 0.00	− 16.7, *p* < 0.00	− 11.7, *p* < 0.00
Adiposities	− 16.0, *p* < 0.00	− 10.3, *p* 0.001	− 16.5, *p* < 0.00	− 21.3, *p* < 0.00	− 12.1, *p* 0.005
Multiple regression					
Multi	Pain	Symptoms	ADL	Sport/Rec	QOL
Heart disease	− 0.9 (− 2.4 to 0.6)	0.5 (− 0.9 to 1.9)	− 2.9 (− 4.3 to − 1.4)	− 4.2 (− 6.5 to − 1.8)	− 1.3 (− 3.2 to 0.7)
Pulmonary disease	− 5.4 (− 7.0 to − 3.9)	− 5.5 (− 7.0 to − 4.0)	− 5.1 (− 6.6 to − 3.6)	− 7.5 (− 9.9 to − 5.1)	− 6.0 (− 8.1 to − 4.0)
Reuma	− 9.5 (− 12.5 to − 6.5)	− 8.5 (− 11.4 to − 5.7)	− 10.2 (− 13.1 to − 7.3)	− 16.5 (− 21.2 to − 11.9)	− 12.8 (− 16.8 to − 8.8)
Osteoporosis		0.9 (− 3.9 to 5.6)	− 5.8 (− 10.6 to − 1.1)	− 4.8 (− 12.8 to 3.2)	− 4.5 (− 11.1 to 2.3)
Stroke	− 3.1 (− 5.3 to − 0.8)	− 3.5 (− 5.6 to − 1.3)	− 3.4 (− 5.6 to − 1.2)	− 3.9 (− 7.4 to − 0.4)	− 3.3 (− 6.3 to − 0.4)
Diabetic disease	− 7.9 (− 10.0 to − 5.7)	− 7.5 (− 9.6 to − 5.5)	− 9.3 (− 11.4 to − 7.2)	− 13.7 (− 17.0 to − 10.3)	− 10.0 (− 12.8 to − 7.1
Adiposities	− 9.6 (− 16.0 to − 3.3)	− 4.6 (− 10.7 to 1.4)	− 8.5 (− 14.7 to − 2.4)	− 9.2 (− 19.5 to 0.9)	− 4.0 (− 12.0 to − 4.6)

### The effect of multi-disease on the pooled HOOS/KOOS/FAOS scores

The one-way ANOVA test showed a significant effect between the number of comorbidity categories and PROM scores: pain: *F*(2, 1810) = 7.8, *p* < 0.001; symptoms: *F*(2, 1850) = 5.9, *p* < 0.01; ADL: *F*(2, 1778) = 15.6, *p* < 0.001; Sport/Rec: *F*(2, 1783) = 7.1, *p* < 0.001; and QOL: *F*(2, 1818) = 4.8, *p* = 0.008.

The post hoc test showed a significant difference between one and three or more comorbidities (*p* < 0.02) and between two and three or more comorbidities (*p* < 0.02), indicating that disease in more than one region of the body negatively influences the HOOS, KOOS, and FAOS subscale scores.

## Discussion

This study demonstrates that patients with medical comorbidities report significantly lower subscale scores on the HOOS/KOOS/FAOS compared to participants without medical comorbidities. The largest difference in scores was observed in the subscales Sport/Rec, ADL, and QOL. These findings underscore the importance of considering comorbidities when interpreting HOOS/KOOS/FOAS in both clinical and research settings.

Diabetes, rheumatological diseases, and obesity exerted the most pronounced negative effects on the HOOS/KOOS/FAOS.

The preset study includes a representative national Danish sample of almost 8000 citizens of whom 24% were registered with medical comorbidity. We compared subscale scores from HOOS/KOOS/FAOS between patients with and without comorbidity and results showed considerable effects, with comorbidities linked to worse HOOS/KOOS/FAOS subscale scores. This study adds to the evidence by analyzing a nationally representative cohort, providing generalizable insights into the broader population.

The change in the pooled HOOS/KOOS/FAOS subscale scores ranged between − 4.6 and − 10.4 comparing citizens with and without medical comorbidity. Considering a threshold of 8–10 points as a rule of thumb for minimal clinically important difference for the HOOS/KOOS/FAOS, results indicate that comorbidity as a binary outcome is at or below the minimal clinically important difference [17]. Results indicated a large heterogenicity in the effect of different medical comorbidity on HOOS/KOOS/FAOS subscales ranging from a low effect of heart disease to a large effect of rheumatic disease. Results from individual medical comorbidity groups showed several groups above the minimal clinically important threshold, indicating the relevance of considering individual medical comorbidities in a clinical setting. Previous studies have primarily focused on the effects of age, sex, BMI, and osteoarthritis on the HOOS/KOOS/FAOS. Despite the known effect of BMI and osteoarthritis, existing literature includes limited information regarding the effect of specific medical comorbidities on the HOOS/KOOS/FAOS [8–10].

Analysis of medical comorbidity from normative representative samples is lacking in existing literature, although such data may be the most viable way to explore the effect on HOOS/KOOS/FAOS.

The effect of medical comorbidity in patients with hip and knee osteoarthritis has been reported with worse HOOS/KOOS subscale scores if medical comorbidity is also present [12, 18]. However, the effect of specific medical comorbidities such as diabetes, COPD or asthma, chronic rheumatological diseases, osteoporosis, stroke, and heart disease has not been reported. Furthermore, several orthopedic diseases may have non-additive effects on the HOOS/KOOS/FAOS subscale scores, making comparison between selected populations with limited value. For example, patients with osteoarthritis may find it difficult to use stairs regardless of the presence of COPD. To the authors’ knowledge, this study is the first to report the effect of specific comorbidities on the HOOS/KOOS/FAOS from a large-scale national representative sample.

Patients suffering from medical comorbidity in the present study were about 10 years older compared to participants without medical comorbidity. One could speculate that being older influences the outcome of HOOS/KOOS/FAOS, exaggerating the effect of medical comorbidity. However, the effects of age and gender are reported from three comparable populations with almost no effect on the HOOS/KOOS/FAOS, supporting the validity of results from the present study [8–10].

Another important finding from the present study is that patients with multi-disease reported lower HOOS/KOOS/FAOS subscale scores. With an increasing number of medical comorbidities, the HOOS/KOOS/FAOS subscale scores decline. These findings are supported by several studies reporting that a greater number of comorbidities is linked with worsening HOOS/KOOS subscale scores in patients with hip and knee osteoarthritis [12, 18].

How are we to use these results? Our findings emphasize the importance of accounting for individual medical comorbidities in the interpretation of HOOS/KOOS/FOAS. Clinicians should consider the potential impact of chronic conditions when evaluating individual patient outcomes or comparing groups in research. For example, a patient with diabetes or obesity may require tailored interventions or support to achieve comparable outcomes to those without such conditions.

In research, incorporating comorbidity data can enhance study design, analysis, and external validity. Adjusting for comorbidities in statistical models or subgroup analyses may improve the accuracy of findings, while stratified reporting can provide actionable insights for diverse patient populations. Furthermore, understanding the effects of comorbidities on outcome variables may include these patient groups in musculoskeletal research and hence increase the external validity.

## Limitations

A limitation to the present study is the retrospective register-based study design, which implies that conclusions regarding causality are impossible. Moreover, the accuracy of the Danish National Patient Register used for data extraction of medical comorbidity may be a limitation. However, registration in the Danish National Patient Register has been required by Danish law since 1978. The Danish National Patient Register is widely accepted as one of the world’s most valid health registries with several decades of follow-up [19]. Obesity is well-known to be associated with worse HOOS/KOOS/FAOS subscale scores [8–10]. The low number of obese citizens reported in the present study may be due to underreporting of obesity as a medical diagnosis in the register. Another limitation may be the response rate of 29%, although comparable to other large-scale register studies. Supplemental analysis between responders and non-responders did not differ on age, gender, and medical comorbidity, minimizing the risk of a strong selection bias. It is not possible to test if non-responders were different on other variables than age, gender, and medical comorbidity.

Other medical comorbidity diagnosis groups may be of influence on the outcome of HOOS/KOOS/FAOS; however, further subgroup analyses were not possible due to the population size.

## Conclusion

This study revealed that patients with medical comorbidity have considerably lower subscale scores on the HOOS/KOOS/FOAS compared to participants without medical comorbidity. Diabetes, chronic rheumatological diseases, and adiposities were observed with the most severe complaints on the HOOS/KOOS/FAOS subscale scores. Such information is highly important in clinical practice when interpreting an outcome derived from these widely used PROMs and in research when HOOS/KOOS/FAOS questionnaires are used to capture outcomes.

## Supplementary Information

Below is the link to the electronic supplementary material.

Supplementary file1 (PDF 28 kb)

## Data Availability

Data are available on request from corresponding author.
