# Supplementary material for: The effect of medical comorbidity on HOOS/KOOS/FAOS: a national register-based cohort study of 7850 representative citizens
Source: Clin Rheumatol. 2025 Feb 22;44(4):1811–8. doi: 10.1007/s10067-025-07372-5 (PMC11993466; doi:10.1007/s10067-025-07372-5)
Supplement: Supplementary file 1 — Supplementary file1 (PDF 28 kb) [file 10067_2025_7372_MOESM1_ESM.pdf]

Supplementary Table 1:

	ICD*
Diabetes	E1*
COPD or astma	J4*
Chronic rheumatological diseases	M05-M14
Osteoporosis	DM8*
Stroke	I6*
Adiposity	E660B, *C,*E,*F,*G,*H
Heart disease	I2*-I7*

* = all subgroups
